# Life Metabolism Travel Prize of 2023

**DOI:** 10.1093/lifemeta/load035

**Published:** 2023-09-05

**Authors:** Life Metabolism Editorial Team

This year (2023) is the second year of our journal, and is the first year for Life Metabolism Travel Prize. The prizes have been generously sponsored by Sable Systems China (sablesystems.cn). The competition this year attracted 36 applications from students and postdocs from across the world. The standard of the entries was really impressive. It was therefore extremely difficult for us to make the final decision. All applications for 2023 Life Metabolism Travel Prize were reviewed by a committee from the editorial board, and ranked based on an anonymized voting procedure. That is, we removed anything that might identify the age, sex, affiliation or nationality of each applicant, and each of the committee independently voted for their favorite six abstracts. So the maximum score anyone could get was 6. We had one person get six votes! And another two got four votes each. These are our winners. We are now pleased to formally announce who they are. They are Alessandra Ferrari at University of California Los Angeles (UCLA), Brandon Chen at University of Michigan, and Meng Yu at Baylor College of Medicine. Congratulations! We also thank all the other applicants for your interest in the competition!

The good news is that the prize will be awarded annually. The idea will be to support any meetings in the next calendar year. We will open the competition in October and we will set the deadline for 8 December. We will then try to make a decision by 1th January, 2024. If you were not successful this time,then note that there is no limit on how many times you can apply, so feel free to apply again when the competition opens shortly.

Let’s hear about the exciting work the winners are doing in their own words.

## Alessandra Ferrari, UCLA, Peter Tontonoz lab

Research interest: cholesterol homeostasis, with a focus on the role played by intestine.


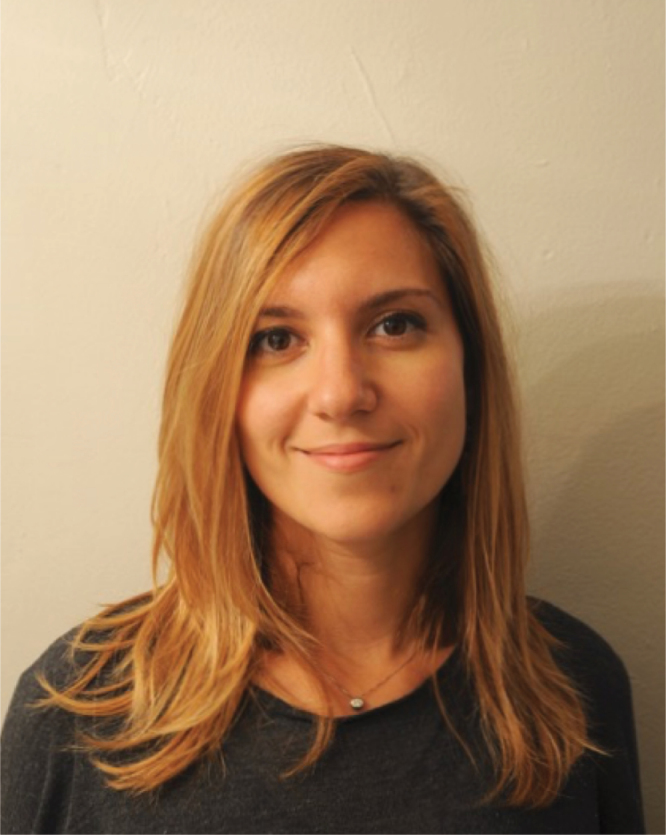


I am a postdoctoral fellow in the laboratory of Peter Tontonoz at UCLA. I have always been fascinated by lipid metabolism, and while in Dr. Tontonoz’s lab, I had the opportunity to study cholesterol homeostasis, with a focus on the role played by intestine. Few years ago, our laboratory identified Asters, a family of protein involved in non-vesicular cholesterol transport in mammalian cells. By using *in vivo* murine models and *ex vivo* systems (intestinal organoids), I found that intestinal Aster-B and Aster-C cooperate with Niemann-Pick C1 Like 1 (NPC1L1) to ensure det-derived cholesterol absorption. Our results showed that NPC1L1 helps in depositing dietary cholesterol into the plasma membrane of enterocyte, expanding the pool of “accessible” cholesterol, available for transfer to endoplasmic reticulum. This transfer is necessary for the subsequent incorporation of diet-derived cholesterol into chylomicrons that are then released into the circulation. Accordingly, I observed that mice with intestinal deletion of Aster-B and Aster-C are protected from diet-induced hypercholesterolemia. The results of my studies also suggested that Aster’s function can be pharmacologically modulated by a small molecule inhibitor, which is able to reduce cholesterol absorption in mice. Overall, this finding suggests that Asters could be a promising candidate for further research and development of pharmacological interventions to regulate cholesterol absorption and potentially treat hypercholesterolemia.

## Brandon Chen, University of Michigan

Co-advised: Costas Lyssiotis lab and Yatrik Shah lab.


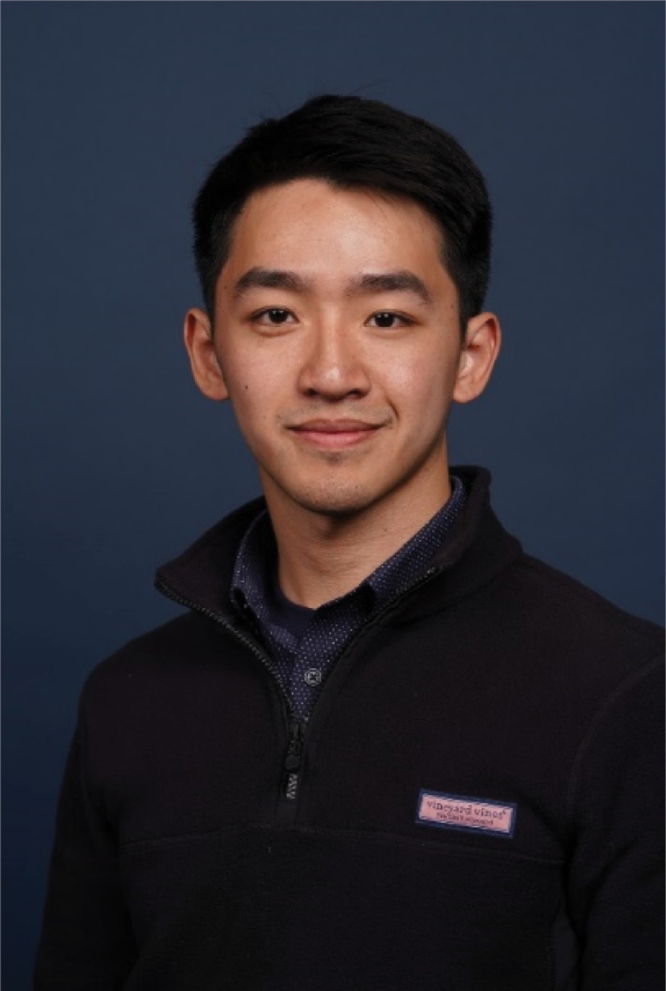


Metabolic reprogramming in cancer is essential to maintain key features such as proliferation, survival, and therapy resistance. A key mechanism for maintaining cellular homeostasis is the compartmentalization of metabolism within organelles, and the alteration of cellular metabolism relies on the coordination and interaction of these organelles. A comprehensive understanding on spatiotemporal coordination of metabolic pathways within a cancer cell is unclear. During my PhD, I became fascinated by the rewiring and coordination of compartmentalized organellar metabolism in tumors to promote cellular fitness. The fine-tuning of metabolism by inter-organelle crosstalk occurs at membrane contact sites, which directly impact metabolite transport, signaling, and organellar function. Among the various inter-organelle interactions, the most abundant interactions occur at endoplasmic reticulum-mitochondria contact sites (ERMCS). ERMCS are formed between proteins from endoplasmic reticulum (ER) and mitochondria and are critical for phospholipid transport, calcium handling, mitochondrial dynamics, and autophagosome formation. However, there is still much to discover about the metabolic signals that promote ERMCS formation, their regulation, and how they facilitate metabolic adaptations. My research focuses on understanding the spatiotemporal regulation of tumor metabolism regulated by ERMCS utilizing cellular, biochemical, and pharmacogenomic approaches.

## Meng Yu, Baylor College of Medicine, Yong Xu lab

Research interest: metabolic effects of retinoic acid (RA) signaling.


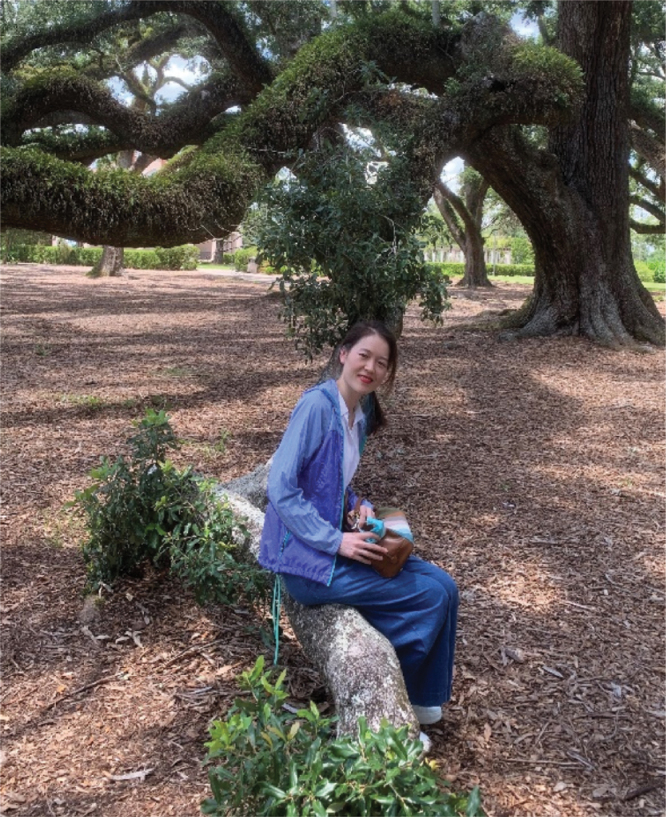


Obesity is a serious global health problem due to its increasing prevalence and strong association with metabolic disorders. The hypothalamus in the brain is essential for energy homeostasis. One of my research directions is about the metabolic effects of RA signaling. Previous reports suggest that RA signaling plays a role in energy balance. RA is the active metabolite of vitamin A and RA signal is mediated by retinoid X receptors. I am exploring the function of retinoid X receptors in hypothalamic neurons on energy homeostasis, which will provide more specific therapeutic targets for obesity. My other direction is neuroendocrine mechanisms for metabolic adaptions during lactation. During lactation, females experience dramatic hormonal and metabolic changes to support high nutrition demand for nursing. Dysregulations of maternal energy homeostasis have detrimental effects on not only offspring, but also moms, including breastfeeding failure and postpartum body weight retention (PPWR). Importantly, PPWR is a potential risk for woman obesity which is understudied. It will provide potential strategies for the woman obesity.

